# A Mathematical Model of Forgetting and Amnesia

**DOI:** 10.3389/fpsyg.2013.00076

**Published:** 2013-02-28

**Authors:** Jaap M. J. Murre, Antonio G. Chessa, Martijn Meeter

**Affiliations:** ^1^Department of Psychology, University of AmsterdamAmsterdam, Netherlands

**Keywords:** amnesia, forgetting, mathematical modeling, hippocampus, cortex, consolidation, Alzheimer’s disease, Korsakoff’s syndrome

## Abstract

We describe a mathematical model of learning and memory and apply it to the dynamics of forgetting and amnesia. The model is based on the hypothesis that the neural systems involved in memory at different time scales share two fundamental properties: (1) representations in a store decline in strength (2) while trying to induce new representations in higher-level more permanent stores. This paper addresses several types of experimental and clinical phenomena: (i) the temporal gradient of retrograde amnesia (Ribot’s Law), (ii) forgetting curves with and without anterograde amnesia, and (iii) learning and forgetting curves with impaired cortical plasticity. Results are in the form of closed-form expressions that are applied to studies with mice, rats, and monkeys. In order to analyze human data in a quantitative manner, we also derive a relative measure of retrograde amnesia that removes the effects of non-equal item difficulty for different time periods commonly found with clinical retrograde amnesia tests. Using these analytical tools, we review studies of temporal gradients in the memory of patients with Korsakoff’s Disease, Alzheimer’s Dementia, Huntington’s Disease, and other disorders.

## Introduction

Since the 1950s, many models of long-term memory and retrograde amnesia have been published that are based on an abstraction of the neuroanatomy, giving a particularly important role to the hippocampus and adjacent medial temporal lobe (MTL) structures (see McClelland et al., 1995; Squire and Alvarez, 1995; Murre, 1996; Meeter and Murre, 2004a, 2005). These models postulate that memories show an initial dependence on the hippocampus that diminishes with time. This process of becoming-independent is often referred to as memory *consolidation* (Meeter and Murre, 2004a). It is typically assumed that repeated reinstatement of a hippocampal-neocortical representation drives this systems-level consolidation process, which authors believe (largely) takes place during sleep (e.g., Stickgold et al., 2000; Stickgold, 2005; Ellenbogen et al., 2006; Marshall and Born, 2007). In the past, we have modeled this process in some detail using a neural network model (Murre, 1996; Meeter and Murre, 2005), demonstrating that such a model can explain many of the characteristics of amnesia and semantic dementia (Meeter and Murre, 2004b). The work presented here can be seen as an abstraction of our earlier work based on neural network models of amnesia (Murre et al., 2007), which also assumed a hippocampus-to-cortex consolidation processes. In this paper we extend this model and apply it to a wider range of data.

Although several models have been able to qualitatively reproduce some data emerging from the study of amnesia, only a few studies presenting quantitatively rigorous treatments have appeared, mostly – like our model – in the context of a neural network model (McClelland et al., 1995; Nadel et al., 2000). If it were possible to capture the shape of retention in various forms of amnesia, this could be used to better validate tests of amnesia and connect parameters at the neural level, such as the severity of a lesion, to behavioral measures, such as the gradient of the retrograde amnesia curve. In this paper, we describe a model of learning and forgetting, the Memory Chain Model, and demonstrate that it can also account for amnesia. The model’s mechanisms are a high-level abstraction of known processes and structures in the brain: a newly learned pattern mobilizes a cascade of mechanisms such as firing neurons, activated neural assemblies, synaptic changes, neural recruitment, and axonal growth (e.g., Milner et al., 1998; McGaugh, 2000). These processes are all able to hold a memory for a certain time period, from ultra-brief to very long.

Although the mechanisms of memory differ vastly in quality and scale, it is our main hypothesis that all neural mechanisms involved in memory share two fundamental characteristics, which form the basis for our mechanism of abstraction: First, a process’s memory strength diminishes over time. Second, as long as a memory has not been lost, it continues to generate or induce more permanent memory processes in a higher-level store. For example, as long as neural assemblies are firing, synaptic enhancement may take place: one process induces another, more permanent process. It is our hypothesis that these two fundamental properties operate on all time scales in roughly the same manner.

If our hypothesis is correct it would explain why forgetting curves can be described by the same shape function, whether ranging over seconds, months, or years, despite very different underlying neural processes (e.g., based on firing neurons, changes in synaptic strength, or growth of entirely new connections). Suppose, for example, that we have two processes, say WM (working memory) and LTP (long-term potentiation in hippocampus). Then, in terms of our assumptions, we have the situation where WM is decaying while trying to “write” its contents to hippocampal LTP, which itself would also decaying exponentially, though at a much slower rate. The question we ask ourselves in this paper is: Given that our hypothesis holds true, what would be the resultant, combined process in terms of retrieving the contents from memory? That is, can we say something about the shape retention curve? As it turns out, the mathematical expressions for such a process can indeed be derived and are well-formed. Moreover, they can be adapted to prevalent memory measures such as cued recall and recognition and the extension to neural systems is straightforward. Furthermore, if we assume that a retention curve is the result of several interacting neural processes, also the pathological curves can be derived and analyzed. These are the memory curves obtained from amnesia patients or experimental animals,. The model, thus, ties neurobiology and pathology to behavior. For this it is crucial that the parameters in the equations correspond in a meaningful and transparent manner to neurobiological or psychological processes or systems, which is what we aim to accomplish in this paper.

The main objective of this paper, thus, is to verify our hypothesis about the uniformity of neural memory processes at different time scales. In order to achieve this we apply the model to amnesia and carry out initial tests by fitting the model to a variety of data sets. Though the model was not developed specifically for amnesia, we will show that without any modifications it can account for the data. We will first review the model, leaving the mathematical details for Appendix. The model is then tested on data from animals and human patients in the Section “Results.” In the Section “Discussion,” we evaluate the implications of the results for consolidation theory.

## Theory

Our model assumes that memory processes can be decomposed into a number of processes that contain memory representations. Processes are system-level abstractions of neurobiological processes and structures. Lifetimes of representations in these memory processes range from milliseconds (extremely short-term processes) to decades (very long-term processes).

A memory representation consists of one or more traces, any of which suffices to retrieve the memory. Such a memory trace could for example be a neural pathway that has been strengthened by LTP so that upon its activation a learned response will be elicited. Such a trace can either encode a rather complete copy of a memory (cf. trace replicas in Nadel et al., 2000) or merely a critical feature (in the sense of feature models, as in Murdock, 1974) that allows retrieval of the entire memory representation.

**Figure 1:**
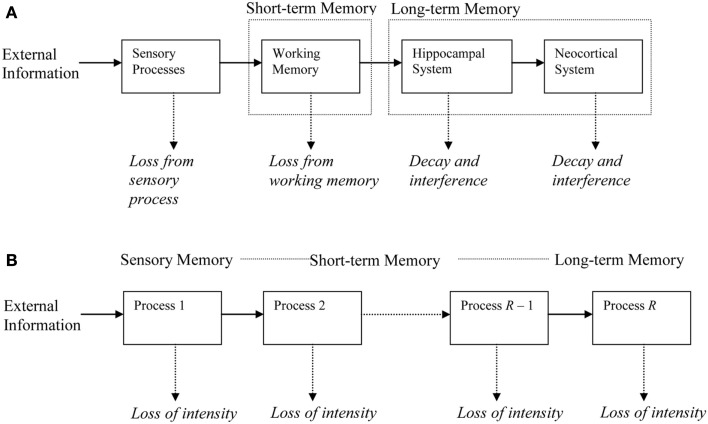
**Illustration of the memory chain**. **(A)** Memory systems at different time scales, with memory decline in each system and induction (generation) of new representations in the next system. **(B)** Abstract representation used in the Memory Chain Model.

During the period of measurement, a newly learned memory will engage one or more of the processes. Processes are chained in a feedforward manner (see Figure 1). Each trace in a process generates traces of its representation in the next higher process, for example through LTP in hippocampus (Abraham, 2003) or neocortex (Racine et al., 1995; Trepel and Racine, 1998). This trace generation is governed by chance, the generation probability being one of the parameters in the model. During initial learning, we assume that the to-be-learned material gradually generates traces in the first process in the chain. A trace has a probability of being lost, for example because it is overwritten by different traces or because of neural noise. All traces in a process share the same loss probability. Once a trace is lost, it can no longer generate new traces in higher processes. Higher processes in the chain have lower decline rates, so that the process sketched here is one of rapidly declining processes trying to salvage their representations by generating traces in more slowly declining processes.

In this paper we will assume that retrieval of a single memory trace in any of the processes suffices for complete recall. The search process initiated by the retrieval cue will typically reactivate only part of a process. This makes memory retrieval stochastic: even if traces are present, it is possible that none will be recovered during attempted retrieval. This is the case if the neural pathways activated by a given memory cue do not manage to connect to any of the memory traces.

One might compare this retrieval process to searching for an unlit candle in a dark apartment using only a flashlight, for example, when the electricity suddenly shuts off during a dark night. Suppose that zero or more candles are randomly scattered around an unfamiliar room. We compare the search for a memory trace in the brain with the search for a candle to light, not knowing beforehand whether any are present. We start pointing our flashlight beam around on the floor randomly until we find a candle, at which point we have accomplished the retrieval. This example highlights four aspects of the search process: (i) A large size of the flashlight beam will speed up the search. This may be compared to a more specific or better memory cue. (ii) The more candles there are, the higher the chances of finding one soon. Thus, if there are more traces in the brain that represent a given memory, chances of retrieval increase. Such an increase is accomplished through additional learning (more memory traces = more candles in the example). (iii) The longer we stumble around with our flashlight, the higher the chance of eventually finding a candle. This suggests that the longer we allow a subject to attempt to retrieve something, the higher the chances of eventual retrieval. (iv) If I have only 1 min to find a candle, I may not find any, even if several are present. Then again, I may get lucky. Thus, in time-limited search, retrieval failures are common even if memory traces are present. Given the same number of memory traces, retrieval may sometimes be successful and at other times it may fail, though more candles will increase chances of success substantially.

Exactly how the different aspects of the neural process interact to produce characteristic shapes of forgetting and amnesia curves is the topic of this paper. A few additional assumptions are necessary to connect the biology to the behavior, for example, how strong a trace must be to still elicit a response. We deliberately abstract from many details of the neurobiological processes (i.e., ignore them), in order to achieve rigorous, systems-level formalization, leaving the role of many of the remaining – possibly highly relevant – details to be explored in other models. After having summarized the results in a few equations, we apply them to a variety of data sets to explore the strengths and weaknesses of the model.

### Formalization

The assumptions introduced above can be translated into a mathematical model that allows the derivation of the shape of learning and forgetting. Appendix gives details of the derivations that are relevant for this paper. We will here limit ourselves to discussing some of the key concepts.

The expected total number of traces is called the *intensity* of the memory. New learning trials add their contribution to the existing intensity (cf. more candles in the example above). Different from the example is that memory traces will start to decline very soon after their formation.

An important neurobiological mechanism of memory trace formation is LTP, which increases due to repeated activation, both in hippocampus (Bliss and Gardner-Medwin, 1973; Abraham et al., 2002) and neocortex (Racine et al., 1995). Longer learning periods and repeated learning trials lead to a proportional increase in intensity by simply adding their contribution, but only up to a point. A biologically plausible model must recognize that neurobiological resources are finite and place limits on the strength and number of synaptic connections that can be formed. There must, therefore, be a maximum to the intensity a memory trace can reach. When this maximum is approached, the learning process saturates and becomes less effective (Huang and Kandel, 1994). With these assumptions, we can describe the shape of the learning curve as well as the advantage of spaced over massed learning in some detail, which we do in a separate paper (Chessa and Murre, 2007).

After learning, various processes may lead to a loss of traces, described by the *decline function*, which describes the decline of intensity after learning as a function of time. Throughout this paper we will assume a constant decay rate, thus arriving at an exponentially declining function. It should be remarked, however, that the exponential decline assumption is not critical for the working of the model, which may also be developed with for example a power function as a decline function, though the resulting equations are more complicated and not all closed-form. Apart from mathematical tractability, there are in our opinion also sound psychological and neurobiological reasons for assuming exponential decay. Our model shares the exponential decline assumption with classic models in memory psychology, for example, the two-process mathematical model by Atkinson and Shiffrin (1968) and the Bower–Lockhart attribute models (Murdock, 1974). Recall data obtained from laboratory experiments that intend to measure short-term memory decline through the classical Brown–Peterson learning and distraction task also support an exponential decline (Peterson and Peterson, 1959). There is, furthermore, evidence at a neural level for exponential decline of LTP within single brain structures (Barnes and McNaughton, 1980; Abraham and Otani, 1991; Abraham, 2003).

The effectiveness of the search processes is determined by the quality of retrieval cues presented to the subject. A good example of this is the study by Wagenaar (1986), who cued his own autobiographical memory by providing himself with one, two, or three memory cues (e.g., about who was present, or when the event took place). In our model, this translates into an increase in the size of the “section of memory” searched in one time unit, increasing the chances of encountering a memory trace (widening the flashlight beam above leads to more floor area covered per minute). In most experiments addressed here, however, the quality of the cues is not varied between conditions and without loss of generality we can set the total size of the cued area per time unit to 1. Cue quality is denoted as *q* (see Table 1 for an overview of symbols and equations).

**Table 1:** **Overview of the equations and symbols used in this paper, assuming *a*_2_ = 0**.

Normal forgetting curve	$p\left(t\right)=1-e^{-\left\{r_{1}\left(t\right)+r_{2}\left(t\right)\right\}}$
	$\;=1-\exp\left(-\mu_{1}e^{-a_{1}t}-\frac{\mu_{1}\mu_{2}}{a_{1}}\left(1-e^{-a_{1}t}\right)\right)$
Ribot gradient	$p_{\text{Ribot}}\left(t\right)=1-e^{-r_{2}\left(t\right)}$
	$\;=1-\exp\left(\frac{-{\text{μ}}_{1}{\text{μ}}_{2}}{a_{1}}\left(1-e^{-a_{1}t}\right)\right)$
Relative retrograde gradient (data transformation)	$rr\left(t\right)=\frac{-\log_{e}\left(1-p_{lesioned}\left(t\right)\right)}{-\log_{e}\left(1-p_{control}\left(t\right)\right)}$
Relative retrograde gradient	$rr\left(t\right)={\left\{\frac{-a_{1}{\left(1-e^{a_{1}t}\right)}^{-1}}{{\text{μ}}_{2}}+1\right\}}^{-1}$

**Symbol**	**Description**

**Free parameters**
μ_1_	Acquired intensity (during learning) of hippocampal/MTL process
μ_2_	Consolidation rate to the neocortical process
*a*_1_	Decline rate of hippocampal/MTL process
*a*_2_	Decline rate of neocortical process (assumed to be 0 here)
λ	Lesion size (0 is no lesion; 1 is full lesion)
**Derived functions**
*p*(*t*)	Recall probability as a function of time *t*
*r*_1_(*t*)	Intensity of the hippocampal (MTL) process
*r*_2_(*t*)	Intensity of the neocortical process
*r*_12_(*t*)	Combined intensity of *r*_1_(*t*) and *r*_2_(*t*)
*rr*(*t*)	Relative retrograde gradient

A special case of cue variation concerns tests of retrograde amnesia for patients. In these tests, the questions for the remote time period are often made easier than for the recent time periods. This can be interpreted as providing better cues for remote time periods, a practice that makes the shape of the retention curves impossible to interpret, each of its points having been manipulated arbitrarily. We return to this point when discussing the relative retrograde gradient (rr-gradient), which aims to remedy this problem.

In the formal model, the effects of learning, storage, and retrieval are multiplied to arrive at the total memory intensity. The intensity increases with learning and decreases with forgetting as a function of time:

$$\begin{array}{llll} \text{memory}\;\text{intensity}\left(\text{time}\right) & =\text{acquired}\;\text{intensity} & & \\ & \;\times \;\text{intensity}\;\text{decline}\;\left(\text{time}\right) & & \\ & \;\times \;\text{cue}\;\text{quality} & & \end{array}$$

Acquired intensity represents the contribution of the learning trial, decline represents the effects of time-dependent storage processes, and cue quality represents the effectiveness of the memory search.

**Figure 2:**
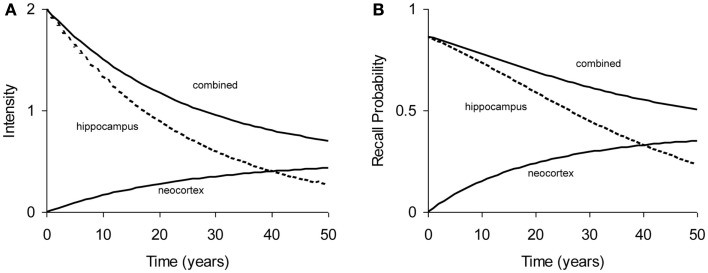
**Example of typical forgetting curve with simulated underlying processes in hippocampal and neocortical stores**. **(A)** Intensity as a function of time. **(B)** Recall probability as a function of time. The curves in **(A,B)** are based on the same parameters.

All experiments analyzed in this paper use probability of recall, *p*(*t*), as the dependent measure, where *t* is the age of the memory: the time elapsed since acquisition of the memory. The relation between memory intensity and recall probability can be described by a simple function: *p*(*t*) = 1 − *e*^−intensity (*t*)^ (see Appendix). Figure 2 shows a typical forgetting function where “hippocampus” process declines rapidly, while the “neocortex” process builds up intensity. In cases of high consolidation rates (e.g., in Stickgold et al., 2000), this may even lead to a temporary increase in total intensity and hence recall probability, but such cases are not modeled in this paper. As the hippocampal process is depleted, the build-up of neocortical process comes to a stand-still, which would eventually turn into a decline (not shown in Figure 2).

**Figure 3:**
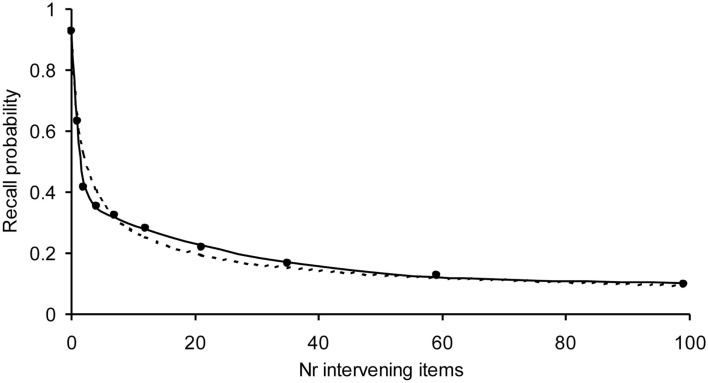
**Example application of the Memory Chain Model to a forgetting curve, with a high number of observations per data point using a three-process recall probability function (solid curve) and the power-law (dotted curve)**. The recall data are word pairs from Rubin et al. (1999). Each data point is based on 1800 observations. The model fitted well on the chi-square test, which becomes more severe with the number of observations (α = 0.55 and *R*^2^ > 0.999). The power-law was rejected by the chi-square test.

We have already tested our model on a variety of experiments with normal subjects demonstrating that our model is able to describe the shape of forgetting and learning. It has also been applied to learning and forgetting of TV commercials and printed advertizements (Chessa and Murre, 2007). An example of a fit to a forgetting curve with 1800 observations per data point is given in Figure 3. Due to the high number of observations, the error bars of the data points would fall within the dots. Despite these high numbers of observations, our model fits these data well, suggesting that we have captured an important aspect of the processes underlying learning and forgetting.

An objection to these types of fitting exercises is that all forgetting curves have roughly the same shape and are thus fitted by a large class of mathematical models (Roberts and Pashler, 2000). In other words, forgetting and learning curves contain only a limited amount of information to constrain models, which is why in this paper we expand the range of application by fitting the model to processes affected by various types of lesions and pathologies. The question is: Can good fits be achieved across conditions and experiments by varying meaningful parameters? Can we, for example, fit the “forgetting” curve of hippocampus-lesioned animals by setting the hippocampus component of the model to zero? Though such fits still do not “prove” that the model is correct, they would be encouraging in that it would seem that model had captured some important elements of memory and the underlying neurobiology. In particular, it would be support in favor of our hypothesis about the common characteristics of generation (induction) and decline of neural memory processes across time scales.

In this paper, we focus on experiments that compare normal forgetting with the effects of lesioning (or disrupting) the MTL and the neocortex. A working hypothesis is that these structures can be identified as two processes of the model. This is a continuation of our earlier work with neural network models of amnesia by us (e.g., Murre, 1996; Murre et al., 2001; Meeter and Murre, 2004a, 2005) and others (e.g., McClelland et al., 1995; Squire and Alvarez, 1995; Nadel and Moscovitch, 1997).

Most of the experiments considered here do not contain data points in very brief retention intervals such as in the seconds or minutes range. If that were the case, we would need early stores like working memory. We will usually assume that these early processes have already run their course and ignore them. So, we consider the hippocampus or MTL as Process 1 and the neocortex as Process 2. It should perhaps be pointed out that this model presents the minimal model that could be applied to these data. In our neural network models (e.g., Meeter et al., 2002; Talamini et al., 2005), we have found it worthwhile to include a third, intermediate process (entorhinal cortex, parahippocampal gyrus). The data considered in this paper, however, are too noisy to allow testing of such higher-order models. We have included studies with different animals, pathologies, procedures, and materials to test the model across a range of data. Most are more or less “classic” data in the field. We are aware that the model should be tested on a much wider range but we consider this work for the future (see Discussion).

Each abstract neural process of the model is characterized by two parameters. The first parameter concerns the rate with which a process fills up with newly generated traces. In this paper, a subscript 1 denotes the hippocampus (or MTL) and a subscript 2 the neocortex (see Table 1). In particular, μ_1_ refers to the intensity acquired during learning, μ_2_ refers to the rate with which the neocortex is filled due to consolidation. The second parameter is the decline rate, which we denote as *a*_1_ and *a*_2_, for the hippocampus and neocortex, respectively.

### The Ribot gradient

Since the work of Ribot (1881) in the nineteenth century, it is known that the temporal gradient in retrograde amnesia, often named “Ribot gradient” in his honor, shows a characteristic pattern with disproportional memory loss for recent time periods. Given our assumption that the hippocampal (MTL) process is damaged in amnesia, the shape of the Ribot gradient can be derived from the Memory Chain Model: it is a retention curve where the contribution of the hippocampal (MTL) process is removed. Below, “Ribot gradient” will refer to such a pathological forgetting curve and “forgetting curve” will refer to the curve of the healthy controls.

In this paper, *r*_1_(*t*) will refer to the intensity of the hippocampal process (as a function of time) and *r*_2_(*t*) to that of the neocortical process (see Table 1). In the Memory Chain Model the total memory intensity is simply the sum of the intensities of the individual processes: *r*(*t*) = *r*_1_(*t*) +  *r*_2_(*t*). A full lesion at time *t*_l_ of the hippocampus translates to removing the contribution of *r*_1_(*t*_l_) from the total intensity *r*(*t*_l_). What remains in such a case is the neocortical intensity, *r*_2_(*t*_l_), which reflects the result of the consolidation process until the lesioning time *t*_l_. It, thus, immediately follows that the shape of the Ribot gradient with a full hippocampal lesion at time *t*_l_ is identical to the expression for *r*_2_(*t*_l_). Tests of retrograde amnesia do not measure intensity directly but they rather measure recall probability. The predicted shape of these test gradients is, therefore, given by $p_{\text{Ribot}}\left(t\right)=1-e^{-r_{2}\left(t_{1}\right)}$ (see Appendix)

If the hippocampus is lesioned at time *t*_l_, then there no more memories will be formed after that. There will also be no more consolidation from hippocampus-to-cortex. That means that if the intensity of a particular memory at the time of the lesion is *r*(*t*_l_), then after that will only follow a decline of the memory intensity with neocortical decline rate *a*_2_, the equation of which is given by $r\left(t_{l}\right)e^{-a_{2}\left(\tau \right)}$ where τ is the time elapsed since the lesion. We have not been able to find data of high enough quality on such post-lesion forgetting curves, though in principle they could be fitted. Hence, we will drop the subscript l in *t*_l_ and continue to write *t* in equations for the Ribot gradient, assuming that in the data considered post-lesion forgetting is negligible.

We often find that neocortical decline (parameter *a*_2_) is close to zero for the material and time periods used in the experiments tested here, for example, because the time period is too short for any neocortical decline to become prominent. Equations for the normal forgetting curve and the Ribot gradient equation are derived in Appendix and listed in Table 1 for the case of no neocortical forgetting and a full lesion of the hippocampal area.

**Figure 4:**
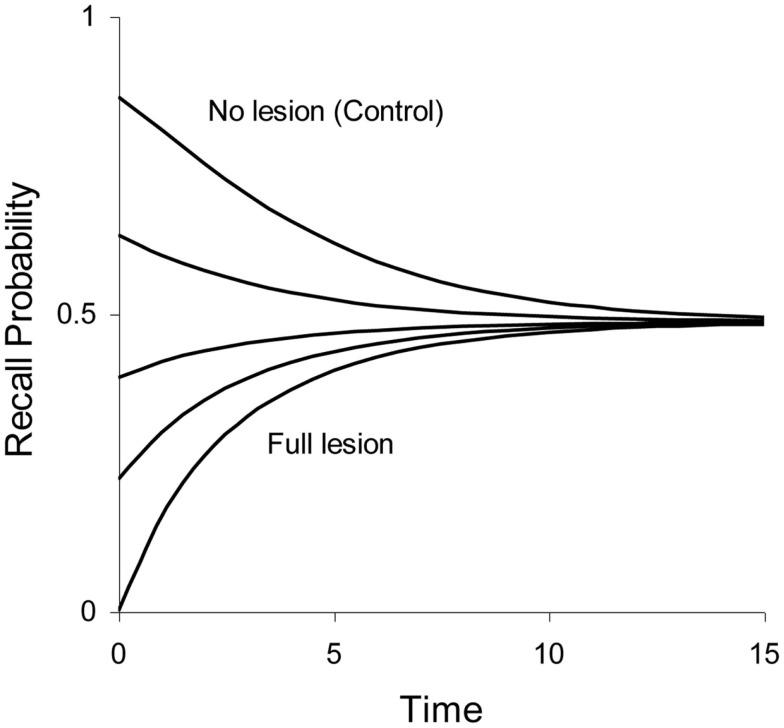
**Illustration of partial retrograde amnesia, from a full lesion to no lesion of Process 1 (hippocampus/MTL)**. Values of the lesion parameter λ were 1.0, 0.875, 0.75, 0.5, and 0.0. The other parameters were μ_1_ = 2.0, *a*_1_ = 0.3, μ_2_ = 0.1, and *a*_2_ = 0 (simulated data with arbitrary time units).

In some lesion studies discussed below, we leave the size of the lesion as a free parameter. The lesion parameter is denoted as λ, with 0 ≤ λ ≤ 1. If the lesion parameter is 0, no lesion is present and if λ =  1 we have a 100% lesion. In case of a partial lesion, the Ribot gradient is equal to $p_{\text{Ribot}}\left(t\right)=1-e^{-\left[\left(1-\lambda \right)r_{1}\left(t\right)+r_{2}\left(t\right)\right]}$. The effects of full and partial lesions of the hippocampal process are illustrated in Figure 4.

### The relative retrograde gradient

As far as we are aware, this is the first time a closed-form expression (i.e., one that can be expressed analytically as a finite sum of well-known functions) is proposed for the Ribot gradient. Unfortunately, there is problem in applying it directly to patient data. This stems from the fact that in tests of retrograde amnesia it is almost never possible to counterbalance items, because by their very nature these items are tied to their time period, some of which will be more memorable than others. Also, items with questions about remote events are typically made easier than recent items (e.g., tests may have recent, rather minor events in national politics and also major, historical events during World War II for the remote time periods). There are good clinical and theoretical reasons for doing this. With a nearly flat but high forgetting curve for the controls (say around 85%), there is a higher chance of uncovering a Ribot gradient for the patient groups because floor effects are diminished for the remote time periods. If this were not done, controls would be near floor for remote time periods and there would be little room to uncover Ribot gradients. When fitting a model, however, this implies that items can only be compared in a pair-wise manner, and not across time periods. The shape of the individual curves is distorted by the manipulation of item difficulty. It also implies that recent and remote items may be of a completely different nature; we will return to this hard-to-evaluate issue in the Section “Discussion.”

Our model offers a way to still use the data of non-counterbalanced retrograde amnesia tests. As we show in Appendix, dividing the intensity (but not the recall probability) of the patient’s curve by that of the control’s curve results in a new curve from which the acquired intensity parameter μ_1_ and the cue specificity parameter *q* are eliminated. The latter parameters are associated among others with how well the items have been learned and how easily they can be retrieved. Removing their effect also removes the distortion. Like the Ribot gradient, the resulting curve is defined over the period before the lesion; it describes the proportion of intensity that has survived (such a curve cannot be obtained by dividing the recall probabilities, because parameters μ_1_ and *q* will not cancel each other out in that case). We call the resulting curve the rr-gradient, because it expresses the shape of the Ribot gradient *relative* to the normal forgetting curve. The shape of the rr-gradient is derived in Appendix and given in Table 1 for the case of a full hippocampal lesion with and without neocortical decline.

**Figure 5:**
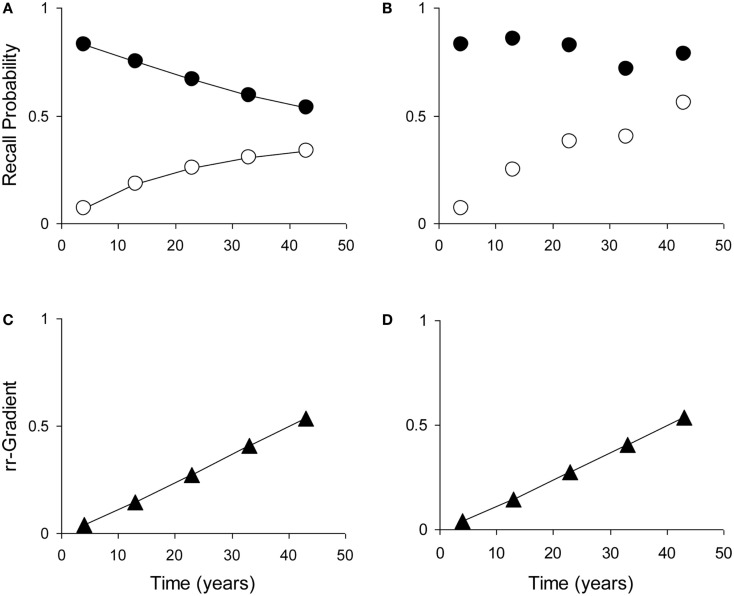
**The relative retrograde gradient remains unaffected by manipulation of item difficulty**. (Illustration with self-generated data points. **(A)** Example forgetting curve (white dots) and Ribot gradient (black dots), generated with the model using μ_1_ = 2, *a*_1_ = 0.04, μ_2_ = 0.01, and *a*_2_ = 0. **(B)** Distorted curve where μ_1_ has been multiplied with (from left to right) 1, 1.4, 1.8, 1.4, and 2. **(C)** Relative retrograde gradient for the undistorted curves. **(D)** Relative retrograde gradient for the distorted curves.

Most tests of retrograde amnesia give us recall probabilities as a function of time elapsed, which is denoted as *p*(*t*). An observed recall probability *p*(*t*) can be transformed into intensity *r*(*t*) by taking −log_e_(1 − *p*(*t*)), where log_e_ is the natural-based logarithm. This data transformation can be carried out on the average test results and is also given in Table 1. One could also take the ratio of the untransformed probabilities *p*(*t*) (e.g., Brown, 2002), but this is less accurate because of the non-linear transformation from underlying trace intensity (which varies over a wide range) to probability (which is constrained between 0 and 1). This means that say a probability of 0.90 does not reflect a trace that is 50% stronger than one that has a response probability of 0.60, but rather one that is more than 150% stronger. Figure 5 illustrates how manipulation of item difficulty leads to distorted forgetting and Ribot gradients. The rr-gradients, however, do not vary.

When lesions of Process 1 (hippocampus/MTL) are not complete, the rr-gradient will not pass through 0 but it will intersect the ordinate. As we show in Appendix, the point of intersection is equal to 1 − λ, where λ is the hippocampal lesion parameter. Thus, if 70% of the hippocampus is lesioned, the rr-gradient will intersect the ordinate at 0.3. It should be remarked that this unlesioned fraction does not necessarily coincide with, say, the volume of remaining tissue and may vary with task difficulty.

**Figure 6:**
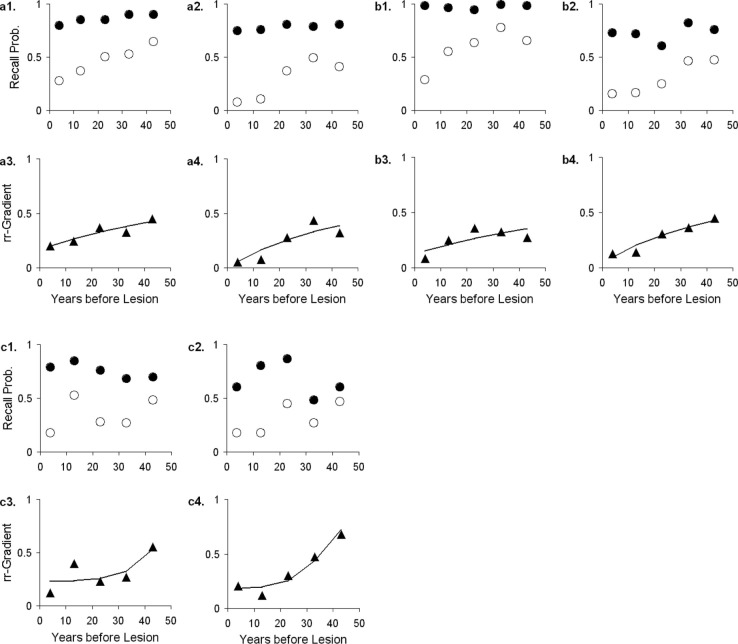
**Data from three studies with Korsakoff patients and controls (Albert et al., 1979; see Studies a–c in our Table 3)**. In each study, panels 1 and 2 represent easy and hard items, respectively. Open circles represent patient data, solid circles controls. Panels 3 and 4 give the relative retrograde gradient for easy and hard items, respectively, shown with triangles. The solid curves are fits by the model, assuming that *a*_2_ = 0.

The rr-gradient is first of all simply a transformation of the data. Figure 5B presents an idealized example of how a jagged forgetting and Ribot curve would be transformed into a smooth rr-gradient. Another characteristic is that when easy and hard questions in a test are plotted separately, they should have the same rr-gradients because they are not affected by item difficulty. In Figure 6, we present data from Korsakoff’s Disease patients and control subjects (Albert et al., 1979), see Studies a–c in our Table 3, divided into easy and hard questions. For each study, the upper panels 1 and 2 show the recall probabilities, while the lower panels 3 and 4 show the empirical rr-gradient (i.e., the transformed data) with a best fitting curve based on our model. The rr-gradients tend to be smoother than the non-transformed curves. Also, the rr-gradients of easy and hard items of one study are more similar than their non-transformed curves.

The rr-gradient allows us to examine the sizable database of human patient studies in retrograde amnesia in a novel manner and make more informed comparisons.

## Results

Unfortunately, data in the neuropsychology of memory is not very suitable for quantitative modeling (Murre, 2002). The number of subjects is often lower than six and the number of observations per data point frequently does not exceed 20. The subjects (patients) often have neuropathologies in addition to the one diagnosed and targeted. These factors give rise to great variability in the empirical curves. Furthermore, the number of data points per curve rarely exceeds five. Such data typically do not constrain models very much: many models may fit them. We attempt here to counteract some of these limitations by fitting our model to a variety of suitable amnesia studies and animal experiments.

For this paper, we selected a number of “classical” data sets from studies that investigate the effects of hippocampal or neocortical afflictions and that include a normal forgetting curve as a control. A restriction was that there be at least four data points per curve, so that we had at least eight data points per study and at least four for the rr-gradient. Studies with merely an ordinal time scale were excluded, unless it was possible to assign plausible time values to the categories. Where appropriate, we will discuss the relevance of the animal results for our understanding of comparable syndromes in human patients.

### Analysis of animal data

#### Forgetting curves and Ribot gradients in retrograde amnesia

**Figure 7:**
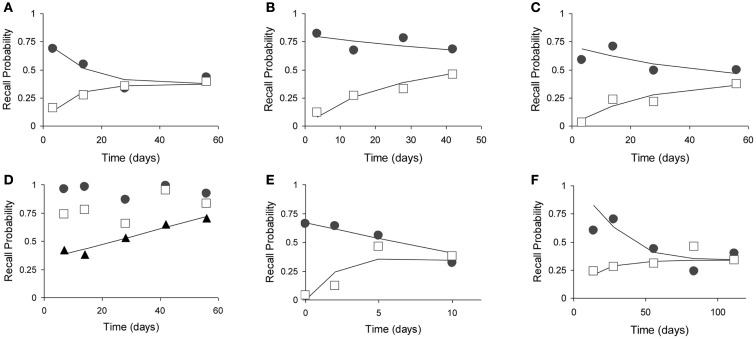
**Fits of the model to six animal experiments on retrograde amnesia**. Experimental animals have lesions to various parts of the MTL (open squares), controls have mock lesions (closed circles). Fitted lines are solid without markers. See Table 2 for further details. **(A)** Using mice with two-choice spatial discrimination (Cho et al., 1993). **(B)** Using mice with two-choice spatial discrimination (Cho and Kesner, 1996). **(C)** Using rats in a contextual fear paradigm (Kim and Fanselow, 1992). **(D)** Using rats (Wiig et al., 1996). Here, the triangles represented the relative retrograde gradient, as in Figure 6. **(E)** Using rats with social learning of food preference (Winocur, 1990). **(F)** Using monkeys in a delayed matching to target task (Zola-Morgan and Squire, 1990).

We selected the six prospective animal studies on retrograde amnesia that include at least four data points per curve (see Figure 7; Table 2). We fitted the pathological and control curves simultaneously with a two-process model. Given the noisy data and the relatively short period of measurement (up to a few weeks or months), we hypothesized that neocortical decline would not yield any measurable effects. We therefore set the decline parameter of the neocortical process *a*_2_ to 0 in all fits. We also assumed that the lesions would be complete rather than partial, allowing us to fix λ at 1. We thus had 8 or 10 data points and three parameters per study: acquired intensity during the training phase μ_1_, hippocampal decline rate *a*_1_, and neocortical consolidation rate μ_2_.

**Table 2:** **Fits to experiments with mice, monkeys, and rats with lesions or transgenic alterations in the indicated structures**.

Study	Experimental design factors					Model				
	P	Time-scale	Task	Lesion	*D*	μ_1_	μ_2_	*a*_1_	*R*^2^	MTL lifetime (days)
Cho et al. (1993)	m	3.5–56 days	Two-choice spatial discrimination	EC	8	1.50	0.00325	0.103	0.94	9.7
Cho and Kesner (1996)	R	3.5–42 days	Spatial discrimination	EC	8	1.60	0.0146	0.0249	0.97	40.2
Kim and Fanselow (1992)	R	3.5–56 days	Contextual fear conditioning	H	8	1.07	0.0149	0.0279	0.93	35.9
Wiig et al. (1996)	R	7–56 days	Visual discrimination	F	5		0.00798	0.0335	0.94**^†^**	29.9
Winocur (1990)	R	0–10 days	Socially acquired food preference	DH	8	1.21	0.124	0.286	0.88	3.5
Zola-Morgan and Squire (1990)	M	14–112 days	Delayed matching to stimulus	H	10	1.78	0.0116	0.0446	0.71	22.4
Frankland et al. (2001)	m*	1–50 days	Contextual fear conditioning	NC	23	0.372	0.519	0.326	0.98	3.1

P, participants; m, mice; m*, genetically altered mice; M, monkeys; R, rats; EC, entorhinal cortex, H, hippocampus, F, fornix, DH, dorsal hippocampus, NC, impaired neocortical plasticity; D, number of data points; parameters are given for time units in days. **^†^**Parameter values are for rr-gradient.

During our analyses, the study by Wiig et al. (1996) gave a very bad fit, with the model explaining less than 20% of the variance (*R*^2^ < 0.20). Inspection of the curves suggested that the time periods were of unequal difficulty, varying in pairs. We contacted the first author, who confirmed that items had indeed not been counterbalanced. The dominant item-effects obscure the true shape of the forgetting and Ribot gradients. We, therefore, calculated an rr-gradient for these data, which removed the item-effects and exposed a much smoother empirical curve (see triangles in Figure 7D).

The fits are summarized in Table 2 and give *R*^2^ values in the range from 0.70 to 0.96. Our model explains 89% of the variance in the data on average. Clearly, the high noise level in the data imposes only weak constraints on the shape of the fitted curves. Nonetheless, we can conclude that the model provides an adequate account for the animal Ribot gradients without adding new parameters to the original model.

#### Forgetting with anterograde amnesia

The Memory Chain Model predicts altered observed forgetting curves when Process 1 (hippocampus) has been lesioned. For short-term forgetting, there cannot be any transfer from working memory to a lesioned hippocampus, so there will be increased forgetting in case of full or (substantial) partial hippocampal lesions.

##### Squire and Zola-Morgan (1991)

**Figure 8:**
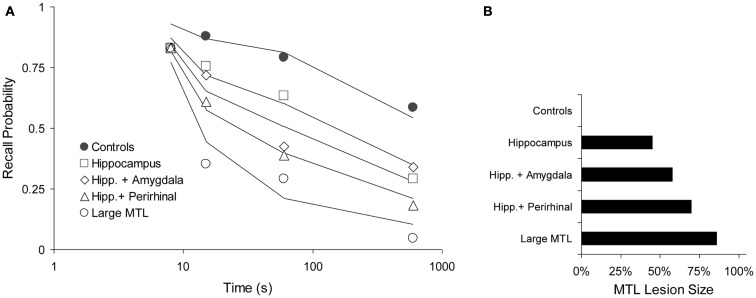
**Forgetting with various levels of anterograde amnesia caused by increasing lesion sizes in a DNMT task (Squire and Zola-Morgan, 1991)**. **(A)** Data (points) and model fit (solid lines). Performance as a function of delay period (logarithmic time scale). **(B)** Relative, functional lesion sizes in the medial temporal lobe (MTL), derived from the working-memory-to-MTL learning rates (see text). Here, 100% would be a full, functional lesion.

One animal study by Squire and Zola-Morgan (1991) is particularly suitable to test our prediction. Monkeys were tested in a delayed non-matching to sample paradigm with delays of 8, 15, 60, and 600 s. Five groups of animals received lesions of the MTL, as follows: I. No lesion (Controls), II. Hippocampus, III. Hippocampus and amygdala, IV. Hippocampus and perirhinal cortex, and V. Large MTL lesion. We fitted these data with a two-process model, assuming that “Process WM” was a form of working memory and Process 1 corresponded with the MTL, as elsewhere in this paper. The model was fitted simultaneously to all data points, leaving only the lesion parameter free for each of the four lesioned curves to reflect the varying sizes of the MTL lesions (For the control curve, we assume no lesion). We thus fitted 20 data points with seven free parameters: acquired intensity in working memory (shared among all curves, μ_WM_ = 5.61), decline in working memory (shared, *a*_WM_ = 0.185), working-memory-to-MTL learning (shared, μ_1_ = 0.0599), and four lesion sizes expressed here as decreased working-memory-to-MTL learning rates, μ_1_, of 0.0329, 0.0252, 0.0180, and 0.0084, for conditions II–V, respectively (see Figure 8B, in which these values have been translated to lesion sizes, relative to the unlesioned value of 0.0599). Although the data are rather noisy, the general pattern is clear enough and it is adequately reproduced by the model, which explains 95.4% of the variance (see Figure 8A).

#### Cortical amnesia

The six animal studies on retrograde amnesia above show how long-term memory fractionates into two neuroanatomically distinguishable memory processes, where Process 1 is the hippocampus (or MTL) and Process 2 is the neocortex. In these studies, Process 1 is lesioned in an experimental group but not in a control group. The opposite pattern could conceivably also occur. Frankland et al. (2001) carried out a forgetting study with genetically manipulated mice in which neocortical plasticity was nearly absent as measured by long-term potentiation. Hippocampal plasticity, however, was intact. The neuropathology of semantic dementia has similar effects on hippocampal and neocortical learning (Murre et al., 2001; Meeter and Murre, 2004b), so that the genetically altered mice (Frankland et al., 2001) can be viewed as a partial animal model of semantic dementia.

We analyzed the first experiment, which used a fear-conditioning paradigm (see Figures 1A,C,E,F in Frankland et al., 2001). A foot shock was paired with a context; after a retention delay the animal’s fear reaction when placed in the experimental context was evaluated. In Experiment 1a, both experimental and control mice (wild-type) were given three foot shocks and evaluated for freezing after retention delays of 1, 3, 10, 17, and 50 days. In Experiment 1b, eight foot shocks were given. In Experiment 1c, control mice that were given one foot shock were compared with experimental mice that were given eight foot shocks. In Experiment 1d, freezing was measured after daily single foot shocks for three consecutive days.

We fitted the data using four parameters: intensity acquired per learning trial (i.e., per shock) μ_1_ = 0.372, hippocampal decline rate *a*_1_ = 0.326, consolidation rate (hippocampus-to-cortex) μ_2_ = 0.519 in wild-type mice and μ_2_ = 0 in genetically altered mice. The multiplication factor used for eight foot shocks was 3.34, meaning that we assume that eight massed foot shocks were as effective as 3.34 shocks under non-massed conditions. We assumed that the cortical decline rate *a*_2_ was zero for the time course of the experiment.

**Figure 9:**
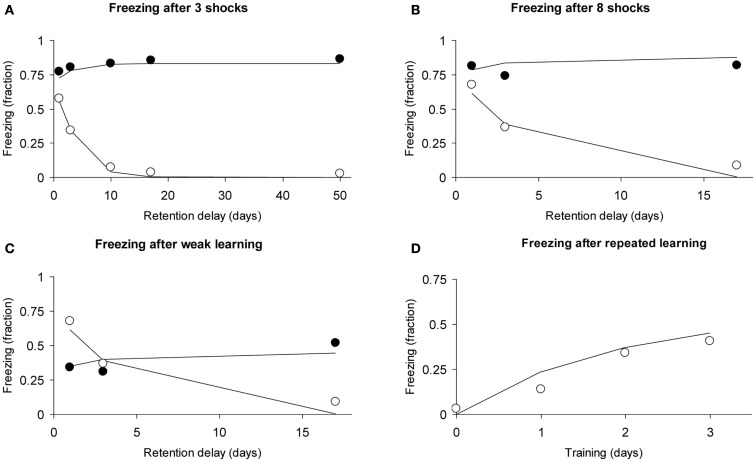
**Observed freezing data (circles) and predicted data (lines) of the study by Frankland et al. (2001) using the assumption of zero consolidation in the experimental condition (see text)**. Open circles refer to experimental subjects (mice), closed circles to controls. **(A)** Forgetting curves after learning with three foot shocks. **(B)** Forgetting curves after learning with eight foot shocks. **(C)** Forgetting curves after learning with one foot shock (controls) and eight foot shocks (experimental). **(D)** Repeated learning in experimental animals receiving one foot shock per day. The observed data are averaged over two conditions (see text for details).

Without learning in the neocortex, retention depends solely on hippocampal decline. The Memory Chain model would therefore predict that the genetically manipulated mice would show abnormally steep forgetting. The data and model fits are shown in Figures 9A–C and meet our expectations. Not only do they validate the qualitative prediction, we also consider the quantitative evaluation of the model’s fit to these data satisfactory, considering we are using a total of only four parameters for all curves.

Frankland et al. (2001) also report a learning curve under repeated learning conditions (daily single foot shocks). Given that we have only a few data points, we will fit the learning curve using a straightforward approach: We simply add the intensities acquired at each learning trial, taking into account forgetting. Intensity for the first trial would be *r*_1_(0), for the second trial, *r*_1_(1) + *r*_1_(0), for the third data trial: *r*_1_(0) + *r*_1_(1) + *r*_1_(2), where the numbers 0, 1, and 2 refer to how many days ago the learning trials had been received. Similarly, the fourth data point was calculated as: *r*_1_(0) + *r*_1_(1) + *r*_1_(2) + *r*_1_(3). In the function *r*_1_(*t*), we used the parameters μ_1_ and *a*_1_ (μ_2_ and *a*_2_ were 0) with the values estimated for the genetically altered mice above.

In the Frankland study (their Figure 1F), one of the two groups of experimental animals had received eight foot shocks 30 days earlier. Our model predicts that after 30 days these will be largely forgotten, because the hippocampal representation will have declined while consolidation to the cortex did not occur. Indeed, in the original data (Frankland et al., 2001), the two curves nearly coincide. We, therefore, compared our predicted learning curve with the average of the two learning curves reported. Our predicted learning curve followed to the data points closely, as can be observed in Figure 9D.

In conclusion, we were able to fit the basic result of the study by Frankland et al. (2001), namely evidence of lack of consolidation to the cortex (Figure 9A). In addition, we could also account for the effects of different learning strengths (Figures 9A–C) and for the effects of repeated learning (Figure 9D). All curves were fitted simultaneously using only four free parameters, explaining 97.6% of the total variance.

### Analysis of human data

Whereas with experimental animals lesions are highly controlled, in amnesia patients lesions are determined by neuropathology. In this section, we will fit our model to studies in which different neuropathologies are investigated, in particular Korsakoff’s Disease, Alzheimer’s Dementia, and Huntington’s Disease.

The areas with greatest damage in Korsakoff’s Disease are ones thought to form one memory system with the hippocampus (Aggleton and Brown, 1999). We therefore modeled this disease by (partially) eliminating the contribution of the hippocampal process. In Alzheimer’s Dementia, hippocampal atrophy is in a later stage of the disease accompanied by diffuse cortical damage, involving loss of synaptic connections and entire neurons. This means that upon retrieval the intensity of the neocortical process is decreased. Patients with Alzheimer’s Dementia were therefore fitted by taking into account both hippocampal lesions and lowering the intensity function of the neocortex by taking (1 − λ_2_)·*r*_2_(*t*), where λ_2_ is the neocortical (functional) lesion size.

Retrograde amnesia in Huntington’s Disease is often tied to retrieval deficits (Deweer et al., 2001). With Huntington’s Disease we therefore assume normal encoding and storage in both processes, but an impaired retrieval process, which is expressed by assuming a cue quality *q* less than 1. The rr-gradient for Huntington patients, their intensity divided by that of the healthy controls, is a constant function: [*r*_1_(*t*) + *r*_2_(*t*)]*q*_H_/[*r*_1_(*t*) + *r*_2_(*t*)] = *q*_H_ < 1, where *q*_H_ is the reduced retrieval cue parameter in Huntington’s Disease. In other words, we expect a flat rr-gradient for Huntington’s Disease that crosses the ordinate at *q*_H_.

All human studies considered here are retrospective. For reasons elaborated above, they can only be fitted with the rr-gradient or a similar relative measure. As with the animal experiments, we set the cortical decline rate to zero, assuming that the effects of long-term cortical forgetting are overshadowed by error sources in the data. With the rr-gradient, μ_1_ and *q* are not used (they cancel out in the derivation when *a*_2_ = 0, see Appendix). Checks with *a*_2_ left free were performed as well, but rarely gave a significant improvement in fit. We usually obtained an adequate fit with three free parameters: *a*_1_ (hippocampal decline rate), μ_2_ (consolidation), and λ (lesion size; 0 is no lesion, 1 is full lesion), with an extra parameter λ_2_ (neocortical lesion size) in case of Alzheimer’s Dementia (though here the hippocampal lesion parameter was shared). We also repeated all fits using a power function instead of an exponential decline, obtaining very similar fits (within a few percentage points of the quoted values) indicating that an exponential decline in a process is not critical for these results. There is analytical evidence that supports these findings: both the Ribot gradient and the rr-gradient have certain fundamental properties that are independent of the choice of the memory decline function (see Appendix).

Summarizing, we predict that Korsakoff patients show rr-gradients that reflect Ribot gradients because the medial temporal system is partially lesioned. Alzheimer’s Dementia patients’ rr-gradients should be similar but lower because of additional neocortical lesions. Huntington’s Disease patients should have flat rr-gradients. We have no clear predictions for the remaining patient groups except that their rr-gradients would also reflect Ribot gradients because of lesions in the MTL.

**Figure 10:**
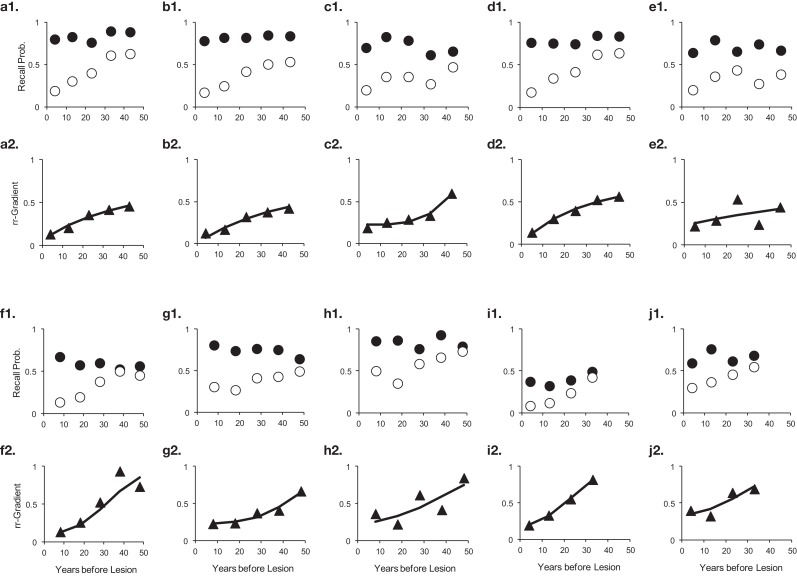
**Ten studies with Korsakoff’s Disease patients and matched controls**. The letters *a*–*j* with each panel correspond to Studies *a*–*j* in Table 3. The panels are presented in pairs, where panel 1 of a pair contains the measured data (solid circles are controls, open circles are patients), and 2 the data transformed to a relative retrograde gradient (always shown as triangles, with the solid line indicating the model fit).

**Figure 11:**
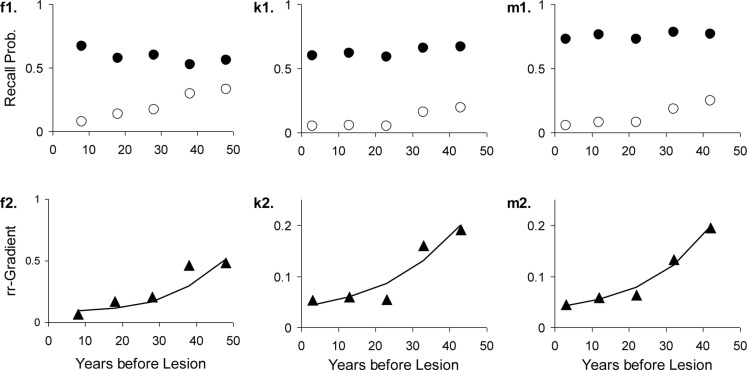
**Three studies with Alzheimer’s Dementia patients and matched controls**. The letters with each panel correspond to those in Table 3. See Figure 10 for further explanation.

**Figure 12:**
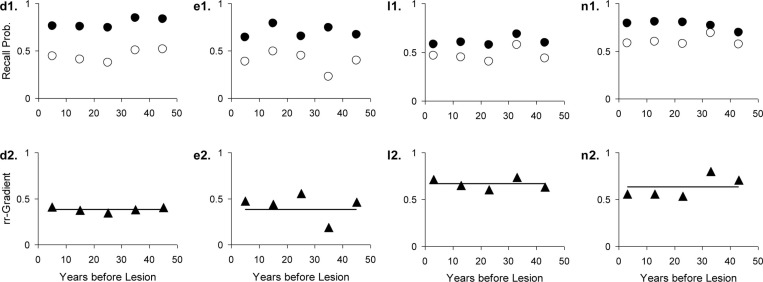
**Four studies with Huntington’s Disease patients and matched controls**. The letters with each panel correspond to those in Table 3. See Figure 10 for further explanation.

**Figure 13:**
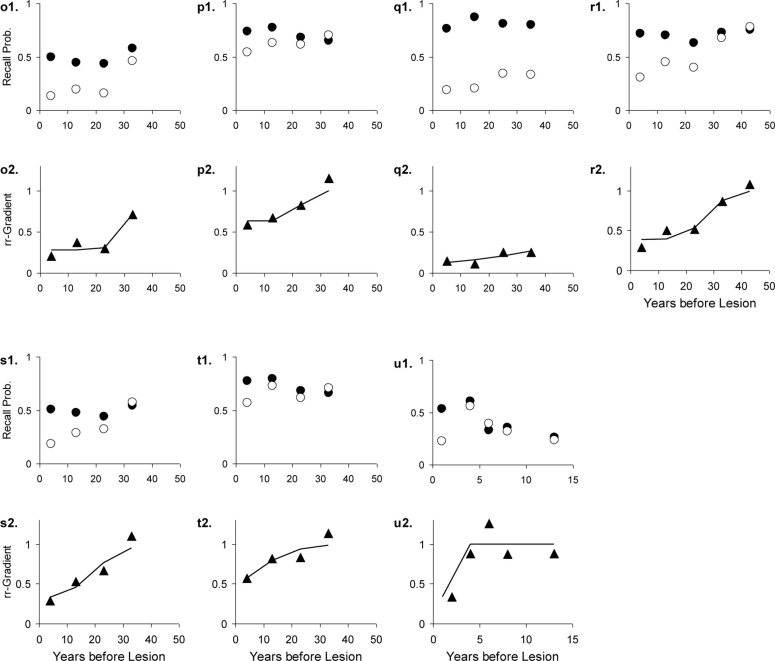
**Seven studies with various patient groups and matched controls**. The letters with each panel correspond to those in Table 3. See Figure 10 for further explanation.

The results are given in Table 3. The data, rr-gradients and model fits are shown in Figures 10–13, for the various pathologies. The model explains about 85% of the data on average (with an average sum of squared differences of 0.035). The results were in line with our predictions. The advantages and limitations of the empirical rr-gradients can be directly observed in these figures: even when the measured curves are quite erratic, the rr-gradients tend to be smooth. When one of the curves approaches floor or ceiling, however, the rr-gradient tends to amplify noise, as the transformation to the underlying intensity then becomes more sensitive to error.

With exception of the study using subjects undergoing electro-convulsive therapy (ECT; Squire et al., 1975), the human rr-gradients all indicate partial lesions, i.e., with a size less than 100%. This indicates either residual functioning of the MTL or a partial dependence of tests on brain areas not affected by the lesion (e.g., involving general knowledge). The animal studies above suggested 100% lesions. We can hypothesize that under laboratory conditions full lesions can be administered with high reliability, whereas patients present with mixtures of partial lesions.

We fitted the Korsakoff and Alzheimer patients of one study (Kopelman, 1989) simultaneously, assuming the same decline and MTL lesion parameters. The fit conforms well to our hypothesis about comparable MTL damage but aggravated neocortical lesions in Alzheimer’s Dementia. Allowing non-shared lesion parameters gave nearly the same results.

Studies k and l (Beatty et al., 1988) in Table 3 use the same tests as Studies m and n, respectively, except that more extensive cues were made available. We can, thus, treat m and n as the easier variants. Separate fits already gave a good agreement of the parameters, as can be verified in the table. Fitting k and m (Korsakoff patients) simultaneously further improved the fit (combined *R*^2^ was 93% variance explained). The same is true for the Huntington patients (Studies l and n).

Huntington’s Dementia was hypothesized to show a flat rr-gradient, a trend that can clearly be observed in Figure 12, although for n2 in Figure 12, it would be possible to fit a non-flat rr-gradient. In the latter case, however, the reliability of the rr-gradient suffers from the fact that the points approach each other in the tail of the curves. The fact that the fitted curve nearly replicates l2 in Figure 12 further reinforces the notion that Huntington’s Disease produces a flat rr-gradient.

A general conclusion is that these human data, though very noisy, are amenable to quantitative analysis if a relative gradient is derived. Moreover, the gradients of the different patient groups conformed to the theoretical expectations.

## Discussion

Above, we have shown how our existing model of learning and forgetting, The Memory Chain Model, can account for a range of amnesia data in a quantitative manner: (i) temporal gradients in mice, rats, and monkeys with various forms of MTL lesions, (ii) increased forgetting gradients in monkeys with progressively large MTL lesions, (iii) increased forgetting gradients in mice that lack neocortical LTP, (iv) the shape of the learning curve of such mice, fitting well without additional parameters, and (v) over 20 data sets from human patients with Korsakoff’s Dementia, Alzheimer’s Dementia, Huntington’s Disease, and other disorders. In most cases, only three free parameters suffice more the model to do an adequate job fitting the data. Though the individual data sets are quite noisy and thus not very constraining, the combination of several such sets offers a more comprehensive test of our model. We conclude that our main hypothesis, about the shared fundamental characteristics of decline of memory traces and their induction in more permanent stores, is not rejected by these fits of the model to these data.

Also, the analyses show that even the noisy neuropsychological data considered here are amenable to quantitative treatment. From the Memory Chain model, we could derive what is probably the first closed-form expression for the Ribot gradient. In the form of the rr-gradient, it also allows retrograde amnesia data to be rid of structural confounds stemming from manipulating test item difficulties.

**Table 3:** **Patient studies of retrograde amnesia examined in this paper**.

Study	Reference	Test		Subjects						Parameters			Fit	
		Topic	Task	C	K	A	H	R	Expl.	*a*_1_	μ_2_	λ	*R^2^*	SSE
a	Albert et al. (1979)	Famous faces	Cued recall	15	11					0.000	0.018	0.941	0.978	0.0017
b	Albert et al. (1979)	Public events	Cued recall	15	11					0.000	0.015	0.946	0.962	0.0025
c	Albert et al. (1979)	Public events	Recognition (3AFC)	15	11					0.148	0.000	0.778	0.967	0.0033
d	Albert et al. (1981)	Famous faces	Cued recall	12	8		13			0.000	0.028	0.988	0.971	0.0035
e	Albert et al. (1981)	Public events	Recognition (3AFC)	12	8		13			0.000	0.008	0.777	0.173	0.1440
f	Kopelman (1989)													
	Korsakoff	News events	Cued recall	16	6					0.077	0.011	0.957	0.857	0.0641
	Alzheimer					8				Shared	0.0024^†^	Shared	0.902	0.0164
g	Parkin et al. (1990)	Famous faces	Cued recall	20	20					0.108	0.001	0.779	0.956	0.0055
h	Parkin et al. (1990)	Famous faces	Cued recall with context	20	20					0.078	0.004	0.779	0.609	0.0936
i	Squire et al. (1989)	Public events	Cued recall	9	7					0.128	0.006	0.831	0.999	0.0003
j	Squire et al. (1989)	Public events	Recognition (4AFC)	9	7					0.096	0.006	0.670	0.793	0.0204
k	Beatty et al. (1988)	Mean of two tests^a^	Cued recall	17		12				0.059	0.001	0.959	0.887	0.0020
l	Beatty et al. (1988)	Mean of two tests^a^	Cued recall	12			12							0.0122
m	Beatty et al. (1988)	Mean of two tests^a^	Cued recall (extra cues)	17		12				0.068	0.001	0.960	0.979	0.0003
n	Beatty et al. (1988)	Mean of two tests^a^	Cued recall (extra cues)	12			12							0.0536
o	Kritchevsky and Squire (1989)	Public events	Cued recall	6				6	TGA^*^	0.361	0.000	0.718	0.905	0.0140
p	Kritchevsky and Squire (1989)	Public events	Recognition (4AFC)	6				6	TGA^*^	0.663	0.000	0.369	0.955	0.0280
q	Reed and Squire (1998)	Mean of eight tests^b^		4				2	P.Enc.	0.034	0.003	0.889	0.683	0.0049
r	Salmon et al. (1988)	Mean of two tests^a^	Cued recall	14				1	Hypoxia	0.256	0.000	0.619	0.941	0.0277
s	Squire et al. (1989)	Public events	Cued recall	8				4	Focal	0.191	0.005	0.689	0.911	0.0376
t	Squire et al. (1989)	Public events	Recognition (4AFC)	8				4	Focal	0.137	0.041	0.521	0.808	0.0338
u	Squire et al. (1975)	TV series	Recognition (4AFC)	16				16	ECT^*^	4.751	0.000	1.000	0.749	0.1122

C, Controls; K, Korsakoff; A, Alzheimer; H, Huntington; R, Remainder; Expl., explanation; P.Enc., Postencephalitis; *within-subjects design; ^†^(1 − λ_2_)μ_2_ (additional neocortical lesion); 3AFC, 3 alternative forced choice; SSE, sum of squared errors. ^a^Famous Faces and Public Events; ^b^Four Recall and Four Recognition.

Several novel predictions can be derived from our model that cannot also be derived from general consolidation theory (Meeter and Murre, 2004a). In fact, consolidation theory only addresses amnesia, and has little to say about normal forgetting or normal learning; one may argue that the theory is *ad hoc* in that respect. We show, however, that consolidation theory leads to an integrated and consistent framework that makes verifiable predictions across a wide range of phenomena. Some of these predictions are qualitative, or at least can be stated verbally without recourse to equations, others are quantitative and concern the precise shape of learning and forgetting curves. We give examples of each.

The rr-gradient generates a prediction, namely that empirical data thus transformed will become insensitive to manipulation of item difficulty. This prediction receives an initial verification in Figure 6. It should be emphasized that if a relative gradient is derived by simply taking the ratio of the probability-correct scores (Brown, 2002), such a manipulation does not generally make the data insensitive to manipulation of item difficulty. Consolidation theory (verbally stated) does not offer such a transformation and it is indeed often hard to interpret retrograde amnesia gradients where the normal controls perform better on the remote items than on the recent ones.

From the fits of the hippocampal decline (forgetting) and cortical consolidation function of the genetically altered mice (Frankland et al., 2001), we can derive quantitative predictions regarding the outcome of a, still to be done, hippocampal lesion experiment: the ensuing Ribot gradient should be described by *r*_2_(*t*) with the parameters found and reported in this paper. This function was not measured directly in their experiment and its precise shape stands as a prediction for follow-up studies. For the Frankland et al. (2001) data we predict that repeated learning trials give rise to a learning curve, which shape we could predict without additional parameters. Such a shape can only be predicted if some underlying measure of intensity (strength) is used with a suitable transformation from intensity to observed behavior (e.g., here, to probability-correct).

A direct prediction from our main hypothesis is that forgetting curves have fundamentally the same shape at both short-term and (very) long-term scales (e.g., forgetting over seconds has the same basic shape as forgetting over decades), and so do learning curves. Our hypothesis also explains *why* this should be the case, namely because forgetting processes share two fundamental characteristics at all time scales (memory decline and induction of memory traces into higher, more permanent processes). Another general prediction of our model is that memory performance can temporarily go up before going down again. No other quantitative model known to us has predicted such reminiscence effects. Yet, they are occasionally reported under circumstances of high or prolonged consolidation (e.g., Stickgold et al., 2000).

One fundamental assumption was that the MTL and neocortex are systems in the Memory Chain Model. This approach is similar to that taken by earlier models (McClelland et al., 1995; Squire and Alvarez, 1995; Murre, 1996). Nadel and Moscovitch (1997) dub this approach the “Standard Theory of Amnesia,” which they reject, proposing instead that memories remain dependent on the hippocampus without consolidation of episodic memories to the neocortex. In their model, partial lesions of the hippocampus will tend to affect older memories less, because these will have built up a stronger representation, while recent memories will not have had this opportunity. Full lesions will always lead to a complete loss of all memories, both recent and remote. Nadel et al. (2000) present both a connectionist model and an analytical model as existence proofs that their assumptions give rise to the characteristics aimed for. Unfortunately, like the model by McClelland et al. (1995), these variants contain unsolvable integrals that must be approximated numerically. They are, therefore, not closed-form expressions and are more difficult to analyze mathematically. Nadel et al. (2000) plot example curves for a range of parameters but these have atypical U-shaped forgetting functions, where memory performance first goes down – as it should – and then goes back up again – which it should not. They also did not include formal fits of the model to data, so it is difficult to assess how it would fare on fits to experimental results. We review and discuss the theory by Nadel and Moscovitch in detail in Meeter and Murre (2004a) and Murre et al. (2001), comparing its merits with consolidation theory and other alternatives.

One of the points of criticism by Nadel and Moscovitch (1997) to consolidation theory is that the MTL seems to hold memories for a very long time, often in the order of decades for humans. We agree with this criticism; the basic finding of long lifetimes in the MTL (Nadel and Moscovitch, 1997) is also found for some of the patient groups considered here. By taking the inverse of the hippocampal decline parameter, $a_{1}^{-1},$ we obtain the expected lifetime of a single trace of a memory in MTL.

For the Korsakoff and Alzheimer data we found expected MTL lifetimes in the order of a decade. In four cases, the model suggested that the MTL remained involved throughout the life of the patients. For these studies, however, higher hippocampal decay rates were obtained if a non-zero neocortical decline was allowed. For ECT, transient global amnesia (TGA), and hypoxia the lifetimes were in the order of 0.2–4 years, suggesting a less widespread pathology. Whereas the human data thus range from a few months to several decades, for the animal data we find MTL expected lifetimes in the range of 3–30 days (see Table 2; also see McClelland et al., 1995).

The wide range in process life times is probably caused by the great variation in to-be-remembered material. Useful comparisons across tasks and species would require a much more extensive theory of the underlying representations than is currently available. In summary, we agree with the authors of the Multiple-Trace Theory (Nadel and Moscovitch, 1997) that the very long life times of memories in the hippocampus/MTL are an oddity, found mainly in the patient fits but not in the fits to animal studies. It is not clear to us, what the implications of this are. On the one hand, a quantitative account can easily incorporate these long-range consolidation processes. On the other hand, what would be the evolutionary mechanism that fosters such extremely slow induction processes, where it takes decades to transfer information from one part of the brain to another? One answer that may offer an interesting compromise is one that elaborates further on the nature of memories after certain periods of time and in different brain areas (Wang and Morris, 2010).

The debate between adherents and skeptics of consolidation theory has continued for over 15 years now. The model presented in this paper is a rather straightforward implementation of some of the main assumptions in consolidation theory, mainly the hippocampus-to-cortex dialog. The model fits the data quite well, but it is possible that a good mathematical model of the Multiple-Trace Theory would fare even better on these data. A problem with deriving such a model, however, is that it assumes within hippocampus consolidation where existing traces multiply and thus lead to a gradual strengthening of certain traces within hippocampus. Such a process of self-consolidation is easily modeled but gives rise to mathematical singularities or “runaway consolidation” (Meeter, 2003). This problem was solved by Nadel et al. (2000) by introducing additional assumptions about trace-dependent time-limited processes but curves shown in the paper are still U-shaped. In our opinion, the Multiple-Trace Theory still awaits a more complete mathematical treatment with fits to data.

The Memory Chain Model currently offers a mathematical framework that is tied to some global aspects of the neurobiology underlying memory. Such a framework allows formal testing of consolidation models with two or more processes, given that suitable data are available. It also allows the development of more powerful clinical tests for diagnosis, for example, through removal of certain item artifacts and decomposition of test results into hippocampal and neocortical memory components. We have recently applied the model to novel tests of retrograde amnesia with ECT illustrating the potential usefulness of the Memory Chain Model in clinical memory experiments (Meeter et al., 2011).

We would like to emphasize that the framework presented here was not originally developed just to explain amnesia, but first and foremost to describe learning and forgetting in normal subjects. Indeed, it has without any modifications been applied to learning and forgetting of TV commercials (Chessa and Murre, 2007) as well as other experiments in short-term and long-term forgetting (e.g., see Figure 3). Here we have shown that the same model can also describe a wide range of amnesia data. This sets it apart from other models of amnesia, nearly all of which have been developed with the aim to (just) explain amnesia. It also distinguishes it from most theories that aim to describe the shape of forgetting, few of which address the possible neurobiological underpinnings. Many such models try to capture the forgetting curve in a simple mathematical function (Wixted and Ebbesen, 1991; Rubin and Wenzel, 1996; Wixted and Carpenter, 2007). Yet, we know that from a neurobiological perspective the forgetting curve is a composite, involving many different processes and structures in dorsolateral prefrontal cortex, hippocampus, and temporal cortex, etc. Any curve-fitting approach based on simple functions such as the power function [i.e., of the shape *p*(*t*) = bt*^−a^*] is, therefore, ultimately doomed to fail on more comprehensive data sets that capture longer time scales, involve certain pathologies or are simply much more precise because of very large samples. Though there is sometimes a good use for such simple functions, we advocate focusing on neurobiological characteristics as a starting point to derive mathematical models that capture the formation and decline of memory parsimoniously.

## Conflict of Interest Statement

The authors declare that the research was conducted in the absence of any commercial or financial relationships that could be construed as a potential conflict of interest.

## Appendix

### Mathematical analyses

#### Forgetting curve

To derive recall probability we note that the number of traces in the region searched during retrieval follows a Poisson distribution (Chessa and Murre, 2007). If we assume that retrieving a single trace suffices for recall, the form of the forgetting function becomes:

$$p\left(t\right)=1-e^{-r\left(t\right)}$$

where *p*(*t*) is the recall probability at time *t* and where *r*(*t*) is the intensity of the memory process at *t*. Here, *t* is the age of the memory since acquisition.

The effects of learning and memory decline are combined in the intensity function *r*(*t*):

$$r\left(t\right)=\left[r_{1}\left(t\right)+r_{2}\left(t\right)+\ldots +r_{R}\left(t\right)\right]q$$

for a multi-process model with *R* processes (in this paper, we usually have *R* = 2). In the equation for *r*(*t*), *q* is the cue strength parameter. Unless mentioned otherwise, *q* will be suppressed (i.e., we take *q* = 1 without loss of generality).

The memory intensity function of the first (hippocampal) process is

$$r_{1}\left(t\right)=\mu_{1}e^{-a_{1}t},$$

which expresses a decline in intensity, assuming a constant decline rate *a*_1_ and an initial intensity (immediately after learning) of μ_1_.

To derive the intensity function of the second (neocortical) process, we hypothesize that there is a rehearsal or consolidation process that generates representations in the neocortical process on the basis of the remaining hippocampal trace. The generation rate is assumed to be proportional to *r*_1_(*t*). While this generation process is still continuing, the content of the neocortical process starts to decline with constant rate *a*_2_. These assumptions give rise to the following expression for neocortical trace intensity *r*_2_(*t*):

$$r_{2}\left(t\right)=\mu_{2}\int_{0}^{t}r_{1}\left(\tau \right)e^{-a_{2}\left(t-\tau \right)}d\tau$$

The integral term expresses an interaction (technically: a convolution) of the generation process from the relatively rapidly declining first (hippocampal) process to the more gradually declining second (neocortical) process. Here, μ_2_ expresses the rate of consolidation from the first to the second process. Straightforward integration and substitution yields:

$$r_{12}\left(t\right)=r_{1}\left(t\right)+r_{2}\left(t\right)=\mu_{1}e^{-a_{1}t}+\frac{\mu_{1}\mu_{2}}{a_{1}-a_{2}}\left(e^{-a_{2}t}-e^{-a_{1}t}\right),$$

where the subscript 12 in *r*_12_(*t*) denotes the intensity function for two processes, i.e., for the composite process that consists of (1) hippocampus and (2) neocortex.

#### Ribot gradient

Process 1 models the hippocampal process, in which we now introduce a partial lesion of size λ, with 0 ≤ λ ≤ 1, 0 meaning no lesion and 1 meaning a full lesion. For the expression of the Ribot gradient we then obtain:

$$r_{\left(1\right)2}\left(t\right)=\gamma \mu_{1}e^{-a_{1}t}+\frac{\mu_{1}\mu_{2}}{a_{1}-a_{2}}\left(e^{-a_{2}t}-e^{-a_{1}t}\right),$$

where γ = 1 − λ. Here, *t* is still the age of the memory and therefore the equation is valid immediately after lesioning. After this, there would forgetting in the neocortex with rate *a*_2_ and no further consolidation from hippocampus (see main text and below). The round brackets of the index in *r*_(1)2_ are used here to indicate a partial lesion of process 1. It can be shown that if *a*_2_ = 0 and γ = 1 − λ = μ_2_/*a*_1_, the Ribot gradient is flat with constant intensity μ_1_μ_2_/*a*_1_. (This implies that in case of a flat, non-zero retrograde amnesia curve, there may well have been a long-term consolidation process operating up until the moment of lesion.) In case of a full hippocampal lesion (γ = 0) and if *a*_2_ = 0, the equation for the Ribot gradient has some interesting properties that are independent of the choice of memory decline function: *(i)* *r*_(1)2_(0) = 0, *(ii)* the derivative for *t* = 0 is equal to μ_1_μ_2_, *(iii)* *r*_(1)2_ is an increasing function, and *(iv)* it has no flex points if μ_1_ > 0.

In the derivation above, we have assumed that relatively little time has elapsed after the hippocampal lesion at time *t* and that *a*_2_ = 0. If this is not the case, the neocortical process will continue to decline with rate *a*_2_, yielding an intensity of $r_{2}\left(t\right)e^{-a_{2}\tau }$ at time *τ* post-lesion, where *r*_2_(*t*) is defined above. In case of a partial lesion, we would have to add to this the continuing effect of consolidation, albeit at a lower rate (e.g., using a rate proportional to γμ_2_: the lesion size multiplied by the original consolidation rate).

#### Relative retrograde gradient

The rr-gradient with a full lesion (signaled with square rather than round brackets around the index of the lesioned process) is defined as *r*_[1]2_/*r*_12_. We obtain a three-parameter curve as follows:

$$rr_{\left[1\right]2}\left(t\right)=\frac{r_{\left[1\right]2}\left(t\right)}{r_{12}\left(t\right)}={\left[\frac{a_{1}-a_{2}}{\mu_{2}}{\left(e^{\left(a_{1}-a_{2}\right)t}-1\right)}^{-1}+1\right]}^{-1}$$

For *a*_2_ = 0, this expression can be simplified to

$$rr_{\left[1\right]2}\left(t\right)={\left[\frac{-a_{1}{\left(1-e^{a_{1}t}\right)}^{-1}}{\mu_{2}}+1\right]}^{-1}$$

In case of partial lesioning of Process 1, we have

$$\begin{array}{l} rr_{\left(1\right)2}\left(t\right)=\frac{r_{\left(1\right)2}\left(t\right)}{r_{12}\left(t\right)}\\ \;=\frac{\gamma r_{1}\left(t\right)+r_{2}\left(t\right)}{r_{1}\left(t\right)+r_{2}\left(t\right)}\\ \;=\gamma rr_{1\left[2\right]}\left(t\right)+rr_{1\left[2\right]}\left(t\right) \end{array}$$

where γ = 1 − λ as above. For *t* = 0, we have *r*_2_(0) = 0, so that rr_(1)2_(0) = γ. In other words, in the case of a partial lesion, the rr-gradient expressed as intensity (not probability) intersects the ordinate at γ. The rr-gradient has other properties that are independent of the choice of memory decline function: (i) it tends to 1 as *t* → ∞; furthermore, if *a*_2_ = 0, then (ii) rr_(1)2_ is strictly increasing, (iii) its derivative for *t* = 0 is equal to λμ_2_, and (iv) it has no flex points if and only if ${r_{1}}^{\prime }\left(t\right)+\mu_{2}r_{1}\left(t\right)>0$ for every *t*, that is, when the induction rate from hippocampus to neocortex is greater than the decline rate in the hippocampus.
